# Relationship between multimorbidity, demographic factors and mortality: findings from the UK Biobank cohort

**DOI:** 10.1186/s12916-019-1305-x

**Published:** 2019-04-10

**Authors:** Bhautesh Dinesh Jani, Peter Hanlon, Barbara I. Nicholl, Ross McQueenie, Katie I. Gallacher, Duncan Lee, Frances S. Mair

**Affiliations:** 1 General Practice and Primary Care, Institute of Health and Wellbeing, College of Medical, Veterinary and Life Sciences, University of Glasgow, 1 Horselethill Road, Glasgow, G12 9LX UK; 2 School of Mathematics and Statistics, University of Glasgow, Glasgow, UK

**Keywords:** Multimorbidity, Mortality, Cancer mortality, Vascular mortality, Condition clusters

## Abstract

**Background:**

Multimorbidity is associated with higher mortality, but the relationship with cancer and cardiovascular mortality is unclear. The influence of demographics and type of condition on the relationship of multimorbidity with mortality remains unknown. We examine the relationship between multimorbidity (number/type) and cause of mortality and the impact of demographic factors on this relationship.

**Methods:**

Data source: the UK Biobank; 500,769 participants; 37-73 years; 53.7% female. Exposure variables: number and type of long-term conditions (LTCs) (*N* = 43) at baseline, modelled separately. Cox regression models were used to study the impact of LTCs on all-cause/vascular/cancer mortality during median 7-year follow-up. All-cause mortality regression models were stratified by age/sex/socioeconomic status.

**Results:**

All-cause mortality is 2.9% (14,348 participants). Of all deaths, 8350 (58.2%) were cancer deaths and 2985 (20.8%) vascular deaths. Dose-response relationship is observed between the increasing number of LTCs and all-cause/cancer/vascular mortality. A strong association is observed between cardiometabolic multimorbidity and all three clinical outcomes; non-cardiometabolic multimorbidity (excluding cancer) is associated with all-cause/vascular mortality. All-cause mortality risk for those with ≥ 4 LTCs was nearly 3 times higher than those with no LTCs (HR 2.79, CI 2.61–2.98); for ≥ 4 cardiometabolic conditions, it was > 3 times higher (HR 3.20, CI 2.56–4.00); and for ≥ 4 non-cardiometabolic conditions (excluding cancer), it was 50% more (HR 1.50, CI 1.36–1.67). For those with ≥ 4 LTCs, morbidity combinations that included cardiometabolic conditions, chronic kidney disease, cancer, epilepsy, chronic obstructive pulmonary disease, depression, osteoporosis and connective tissue disorders had the greatest impact on all-cause mortality. In the stratified model by age/sex, absolute all-cause mortality was higher among the 60–73 age group with an increasing number of LTCs; however, the relative effect size of the increasing number of LTCs on higher mortality risk was larger among those 37–49 years, especially men. While socioeconomic status was a significant predictor of all-cause mortality, mortality risk with increasing number of LTCs remained constant across different socioeconomic gradients.

**Conclusions:**

Multimorbidity is associated with higher all-cause/cancer/vascular mortality. Type, as opposed to number, of LTCs may have an important role in understanding the relationship between multimorbidity and mortality. Multimorbidity had a greater relative impact on all-cause mortality in middle-aged as opposed to older populations, particularly males, which deserves exploration.

**Electronic supplementary material:**

The online version of this article (10.1186/s12916-019-1305-x) contains supplementary material, which is available to authorized users.

## Background

Multimorbidity, the presence of two or more long-term conditions (LTCs), is a global health challenge and an international research priority [1]. The prevalence of multimorbidity varies according to the definition and method of classification used, characteristics of the cohort under study (such as age, sex and socioeconomic status) and country of study [2, 3]. The presence of multimorbidity has been associated with poor quality of life and poor health outcomes, including higher mortality risk [4–6]. However, there are many evidence gaps in understanding the relationship between multimorbidity and mortality, for example, cancer and vascular mortality are the top two causes of mortality but the potential impact of multimorbidity on these outcomes has not been investigated [7]. Additionally, the role of the type of LTCs and their combinations in risk prediction of mortality remains unclear.

A systematic review of 39 studies including > 70 million patients found that demographic factors such as age, gender and socioeconomic status were the most important determinants of multimorbidity [2]. However, the impact of demographic factors on the relationship between multimorbidity and mortality has not been adequately examined. The majority of studies investigating the role of multimorbidity in predicting mortality have focussed on elderly populations, typically those over 65 years of age [6, 8–12]. Some studies have attempted to investigate the relationship between multimorbidity and mortality in adults from all age groups; however, there are significant research gaps as these studies did not account for possible variations in this relationship across different age groups and had a short follow-up duration of 1–3 years [13–15]. The recent Academy of Medical Sciences report highlights understanding the impact of multimorbidity in younger age groups as a key research gap [1]. Multimorbidity is more common in those from socioeconomically deprived backgrounds and is generally reported to also be more prevalent in women [1, 2]; however, the impact of gender and socioeconomic status on mortality in multimorbidity has received less attention. While a number of previous studies have adjusted for the effects of sex and socioeconomic status on the association between multimorbidity and mortality [6, 9, 10, 12, 14, 15], only two studies have examined this association across different gradients of socioeconomic status and found that the effect of multimorbidity remained consistent across different socioeconomic groups [16, 17]. A syndemic approach has been proposed to understand multimorbidity, where the emphasis is to understand the context in which illnesses are experienced, including personal circumstances [1, 18]. Demographic factors are likely to be important contextual factors in studying the impact of multimorbidity. This study aims to address the evidence gap by utilising the UK Biobank, a large cohort of over half a million middle to older aged adults, to examine the relationship between multimorbidity (number and type of LTCs) and all-cause, cancer and vascular mortality and the influence, if any, of demographic factors on the relationship between multimorbidity and mortality.

## Methods

### Study design and participants

This is a prospective population-based cohort study which included 502,640 participants enrolled in the UK Biobank from 22 different assessment centres across England, Scotland and Wales between 2006 and 2010. Individuals were invited to participate on a voluntary basis if they lived within 25 miles of a UK Biobank assessment centre and were registered with a GP. All participants gave informed consent for data provision and linkage. The UK Biobank has full ethical approval from the NHS National Research Ethics Service (16/NW/0274). A self-reported detailed account of sociodemographic, lifestyle and medical information was collected from all participants recruited to the study.

### Procedures

All participants reported their health conditions at the time of study recruitment. The physical and mental health conditions reported by participants were organised into a list of 43 long-term conditions (LTCs) based on previously published literature on multimorbidity (please see Additional file 1: Table S1) [19, 20]. Multimorbidity was classified based on LTC count into no LTCs, 1 LTC, 2 LTCs, 3 LTCs, ≥ 4 LTCs. In the main analysis, socioeconomic status was classified based on Townsend score (a measure of deprivation in the UK) [21]. A Townsend deprivation score calculated using the participant’s home postcode, based on the preceding national census output areas, was provided; a higher score implied higher levels of socioeconomic deprivation. Smoking status was divided into two categories: non-smokers and previous/current smokers. Alcohol consumption was a categorical variable based on the self-reported frequency of alcohol intake: never or special occasions only, one to three times a month, one to four times a week and daily or almost daily. Physical activity was self-reported and classified as none (no physical activity in the last 4 weeks), low (light ‘do it yourself (DIY)’ activity only in the last 4 weeks), medium (heavy DIY and/or walking for pleasure and/or other exercises in the last 4 weeks) and high (strenuous sports in the last 4 weeks) [22]. Body mass index (BMI) calculated from anthropometric measurements at the baseline assessment was classified as per WHO classification into < 18.5, 18.5–24.9, 25–29.9, 30–34.9, 35–39.9 and ≥ 40 kg/m^2^ [23].

### Clinical outcomes

The baseline assessment centre data were linked to national mortality records by the UK Biobank data analysts. The three outcomes studied were all-cause mortality, vascular mortality and cancer mortality. Vascular and cancer mortality are the top two causes of mortality in the UK [7]. The follow-up period ended between November 2015 and January 2016, depending on different assessment centres across the UK. Length of follow-up was a median duration of 7 years (interquartile range 76–93 months). We utilised ICD-10 primary cause of death classifications for defining vascular deaths (ICD-10 codes beginning with ‘I’) and cancer deaths (ICD-10 codes beginning with ‘C’) [24].

### Statistical analysis

Participants with complete data on self-reported LTCs and mortality status were eligible for inclusion in the analysis. The distribution of multimorbidity across various demographic and health-related behaviour characteristics were described using mean and standard deviation for continuous variables and percentages for categorical variables. Survival plots were used to compare cumulative all-cause mortality rates between participants in the four LTC categories (0 LTCs, 1 LTC, 2 LTCs, 3 LTCs, ≥ 4 LTCs). Cox’s proportional hazards regression modelling, using age as the underlying time variable, was utilised to examine the relationship between the number of LTCs and all-cause mortality. The time variable was truncated for survival plots at 76 years due to the smaller number of participants beyond this point. Results were presented in the form of hazard ratios (HR) with 95% confidence intervals (CI), adjusted for confounding variables (sex, socioeconomic status (based on Townsend score), smoking and alcohol status, physical activity levels and BMI). The above analysis was repeated using vascular deaths and cancer deaths as outcome variables, by running separate cause-specific regression models to account for competing risks between the two different causes of death. In each cause-specific model, events due to alternative causes were treated as censored [25]. For example, if a participant died of vascular causes, they were censored from the regression model for cancer death as an outcome. The cumulative incidence function (CIF) was used to create cause-specific mortality plots for each category of the LTC count (no LTCs, 1-LTC, 2 LTCs, 3LTCs, ≥ 4 LTCs). The total number of participants included in the survival analysis models (unadjusted and adjusted) varied according to the completeness of the putative confounding variables, and all missing data were excluded from regression modelling; however, the proportion of missing data was relatively small, ranging from zero to 2.4%.

Next, we examined the role of the type of LTCs in risk prediction of clinical outcomes. We considered three separate predictors: previous history of cancer (no/yes), number of cardiometabolic conditions (0, 1, 2, 3, ≥ 4) and number of non-cardiometabolic conditions (0, 1, 2, 3, ≥ 4). Hypertension, coronary heart disease, peripheral vascular disease, atrial fibrillation, diabetes, heart failure, previous stroke or transient ischaemic attack were defined as cardiometabolic conditions. The rest of the LTCs in the list of 43 LTCs described above, excluding cardiometabolic conditions and cancer, were defined as non-cardiometabolic conditions. These three predictors (cardiometabolic, non-cardiometabolic and cancer) were included within the same model to assess their respective impact on clinical outcomes. Each of the three clinical outcomes (all-cause, cancer and vascular mortality) was modelled separately as previously described.

We then examined the effect of individual combinations of LTCs on all-cause mortality. It was not feasible to test for all possible combinations of LTCs (for example, in the 4 or more LTC category, there are 123,410 possible combinations of 43 LTCs). We therefore restricted generating combinations to the top 25 LTCs with the greatest individual risk of mortality in the whole cohort. Analyses were stratified using the three multimorbid categories, based on LTC count (2, 3, ≥ 4). Additionally, a minimum of 20 subjects per variable has been regarded as a standard requirement for multivariable regression models; hence, we excluded those LTC combinations which had less than 20 observations [26]. In each of these LTC count categories, the HRs with 95% CI for the top 10 LTC combinations with the largest effect sizes on all-cause mortality risk were reported. Participants with no LTCs were used as the reference group for regression models, and all models were adjusted for sex, socioeconomic status (based on Townsend score), smoking and alcohol status, physical activity levels, and BMI.

In the final section, we examined the interaction of demographic factors (age, sex and socioeconomic status) with LTC categories in the risk prediction of all-cause mortality. Age was divided into three categories: 37–49, 40–59 and 60–73. Socioeconomic status was divided into five categories based on the five quintiles of Townsend score: 0–20 (most affluent), 20–40, 40–60, 60–80 and 80–100 (most deprived). Two separate multivariable Cox’s proportional hazards regression models were utilised to study the relationship between LTCs and all-cause mortality, stratified by (a) age and sex and (b) socioeconomic status, respectively. Results were presented in the form of adjusted HRs with 95% CI, adjusted for smoking and alcohol status, physical activity levels, and BMI at baseline.

All statistical analysis was conducted using R software [27]. Three members of the team independently checked all statistical analyses (BJ, PH and DL).

### Sensitivity analyses

The prediction model for all-cause mortality was repeated in sensitivity analyses. The LTC count was reconstructed using LTC defined on using ICD-10 diagnostic records of hospitalisation events prior to study recruitment instead of self-reported history at the time of recruitment [24]. We used the ICD-10 diagnostic codes for *N* = 43 LTCs, as described above, and searched for all hospital recorded discharge diagnoses prior to the study recruitment (prior to the start of the follow-up period).

## Results

### Patient characteristics

In total, *N* = 500,769 participants provided information on LTCs and were successfully linked with mortality status and included in this analysis. Most participants (*N* = 328,176 (65.5%)) reported one or more LTC at baseline. A total of 163,705 participants (32.7%) reported having only one LTC; 95,226 participants (19%) reported having two LTCs; 43,120 participants (8.6%) reported having three LTCs and 26,125 participants (5.2%) reported having ≥ 4 LTCs. Table 1 shows the baseline characteristics of the four LTC groups and the overall study population.

**Table 1:** Relationship of multimorbidity with demographics and health-related behaviour at baseline. *N* = 500,769

	No LTCs *N* = 172,593 (34.5%)	1 LTC *N* = 163,705 (32.7%)	2 LTCs *N* = 95,226 (19%)	3 LTCs *N* = 43,120 (8.6%)	≥ 4 LTCs *N* = 26,125 (5.2%)	Overall *N* = 500,769
Age; missing values *n* = 0						
Age in years-mean (SD)	54.0 (8.1)	56.6 (8.0)	58.5 (7.6)	59.7 (7.2)	60.3 (6.9)	56.5 (8.1)
Sex; missing values *n* = 0						
Male	79,947 (46.3%)	75,240 (46.0%)	43,448 (45.6%)	19,170 (44.5%)	10,695 (40.9%)	228,500 (45.6%)
Female	92,646 (53.7%)	88,465 (54.0%)	51,778 (54.4%)	23,950 (55.5%)	15,430 (59.1%)	272,269 (54.4%)
Socioeconomic status based on Townsend Score; missing values *n* = 626 (0.13%)						
Townsend score-mean (SD)	− 1.5 (3.0)	− 1.4 (3.0)	− 1.2 (3.1)	− 0.9 (3.3)	− 0.4 (3.4)	− 1.3 (3.1)
Smoking status; missing values *n* = 2794 (0.56%)						
Never	102,365 (59.7%)	90,168 (55.3%)	48,593 (51.3%)	20,443 (47.7%)	11,220 (43.3%)	272,779 (54.8%)
Current or previous	69,164 (40.3%)	72,780 (44.7%)	46,130 (48.7%)	22.427 (52.3%)	14,695 (56.7%)	225,196 (45.2%)
Alcohol status; missing values = 1345 (0.27%)						
Never or special occasions only	26,451 (15.4%)	29,356 (18.0%)	20,995 (22.1%)	11,750 (27.3%)	9506 (36.5%)	98,058 (19.6%)
1–3 times/month	18,797 (10.9%)	17,898 (11.0%)	10,760 (11.3%)	5177 (12.0%)	3075 (11.8%)	55,707 (11.2%)
1–4 times/week	91,136 (53.0%)	81,473 (49.8%)	44,021 (46.3%)	18,080 (42.0%)	9423 (36.2%)	244,133 (48.9%)
Daily or almost daily	35,541 (20.7%)	34,670 (21.2%)	19,260 (20.3%)	8017 (18.7%)	4038 (15.5%)	101,526 (20.3%)
Body mass index (BMI); missing values *n* = 3027 (0.6%)						
< 18.5	1033 (0.6%)	833 (0.5%)	438 (0.5%)	177 (0.4%)	128 (0.5%)	2609 (0.5%)
18.5–24.9	68,186 (39.8%)	51,875 (31.8%)	23,838 (25.2%)	8733 (20.4%)	4330 (16.8%)	156,962 (31.5%)
25–29.9	73,953 (43.2%)	71,945 (44.1%)	40,943 (43.2%)	17,501 (40.9%)	9241 (35.7%)	213,583 (42.9%)
30–34.9	22,261 (13%)	28,672 (17.6%)	20,535 (21.7%)	10,699 (25.0%)	7044 (27.2%)	89,211 (17.9%)
35–39.9	4555 (2.7%)	7339 (4.5%)	6434 (6.8%)	3937 (9.2%)	3255 (12.6%)	25,520 (5.1%)
≥ 40	1323 (0.7%)	2359 (1.5%)	2524 (2.6%)	1780 (4.1%)	1871 (7.2%)	9857 (2.0%)
Physical activity; missing values *n* = 6970 (1.39%)						
High	24,815 (14.6%)	16,443 (10.2%)	6239 (6.7%)	1837 (4.3%)	679 (2.7%)	50,013 (10.1%)
Medium	132,926 (78.3%)	130,709 (80.7%)	76,505 (81.3%)	33,690 (79.2%)	18,506 (72.7%)	392,336 (79.5%)
Low	4655 (2.7%)	5665 (3.5%)	4173 (4.4%)	2421 (5.7%)	1931 (7.6%)	18,845 (3.8%)
None	7463 (4.4%)	9027 (5.6%)	7185 (7.6%)	4590 (10.8%)	4340 (17%)	32,605 (6.6%)
Presence of at least 1 cardiometabolic condition	Not applicable	52,592 (32.1%)	52,807 (55.4%)	29,833 (69.2%)	20,957 (80.2%)	156,189 (32.2%)
Presence of previous cancer	Not applicable	12,744 (7.8%)	12,509 (13.1%)	7574 (17.6%)	5795 (22.2%)	38,622 (7.7%)

*LTCs* long-term conditions, *SD* standard deviation, *cardiometabolic conditions* hypertension, coronary heart disease, peripheral vascular disease, atrial fibrillation, diabetes, heart failure, stroke or transient ischaemic attack

### Multimorbidity and mortality

**Fig. 1:**
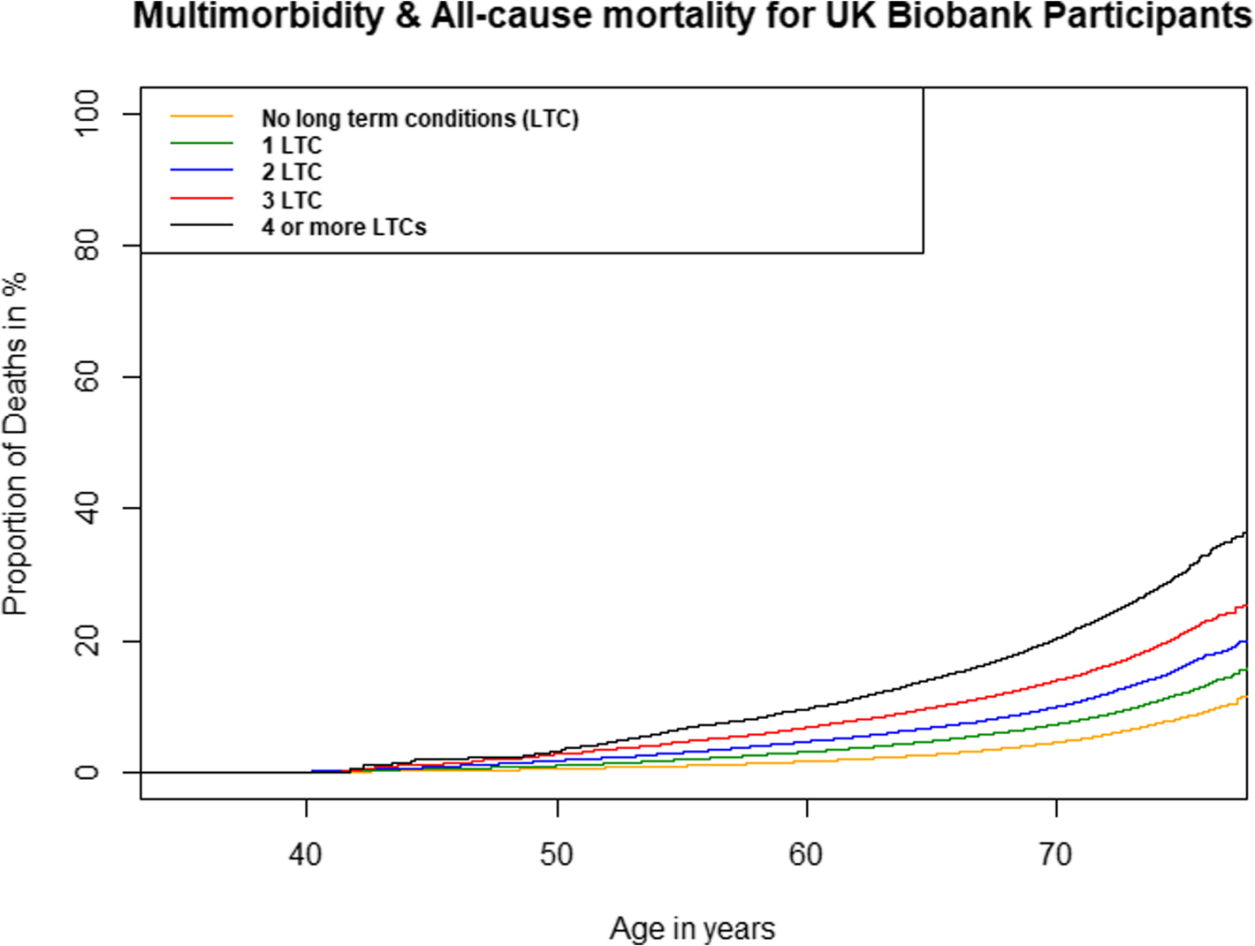
Cumulative survival plot showing the probability of all-cause mortality among the UK Biobank participants with different levels of multimorbidity. *N* = 500,769 UK Biobank participants; *LTCs* long-term conditions

At the end of the follow-up period, 14,348 participants (2.9%) had died; the mean age for those who died was 61.3 years (61.7 years for males, 60.7 years for females). At the end of the follow-up period, 2408 participants (1.4%) in the no LTC group had died, 4147 participants (2.5%) in the 1 LTC group had died, 3555 participants (3.7%) in the 2 LTC group had died, 2213 participants (5.1%) in the 3 LTC group had died and 2025 participants (7.75%) in the ≥ 4 LTC category had died. The respective cumulative mortality curves for participants with higher numbers of LTC had a steeper gradient than those of participants with no LTC at baseline, while participants with ≥ 4 LTC had the highest mortality rate throughout the follow-up period (see Fig. 1). The number of LTCs reported by participants at baseline had a strong association with all-cause mortality over the follow-up period with a dose-response relationship observed in both unadjusted and fully adjusted Cox’s regression analysis (Table 2). In the fully adjusted analysis, participants with 1 LTC were approximately 1.5 times more likely to die compared to participants with no LTC (HR 1.46, 95% CI 1.38-1.54), while participants with ≥ 4 LTC were nearly three times more likely to die than participants with no LTC (HR 2.79, CI 2.61- 2.98). The full results for the regression model in Table 2 are presented in Additional file 1: Table S2.

**Table 2:** Multimorbidity and all-cause mortality over 7-year median follow-up: Cox’s regression analysis

*N* = 500,769	7-year cumulative mortality	Unadjusted number of events = 14,348	Adjusted* (missing values *n* = 12,045, 2.4%); number of events = 13,570
		Hazard ratios (95% confidence intervals)	
No LTC *N* = 172,593	2408 (1.4%)	1	1
1 LTC *N* = 163,705	4147 (2.5%)	1.52 (1.45–1.60)	1.46 (1.38–1.54)
2 LTC *N* = 95,226	3555 (3.7%)	1.98 (1.88–2.08)	1.77 (1.68–1.87)
3 LTC *N* = 43,120	2213 (5.1%)	2.53 (2.39–2.68)	2.14 (2.01–2.28)
≥ 4 LTC *N* = 26,125	2025 (7.8%)	3.72 (3.50–3.95)	2.79 (2.61–2.98)

Age as the timescale for both analyses. *LTCs* long-term conditions. *Adjusted for sex, socioeconomic status based on Townsend score, smoking status, alcohol status, body mass index and physical activity levels reported at baseline

**Fig. 2:**
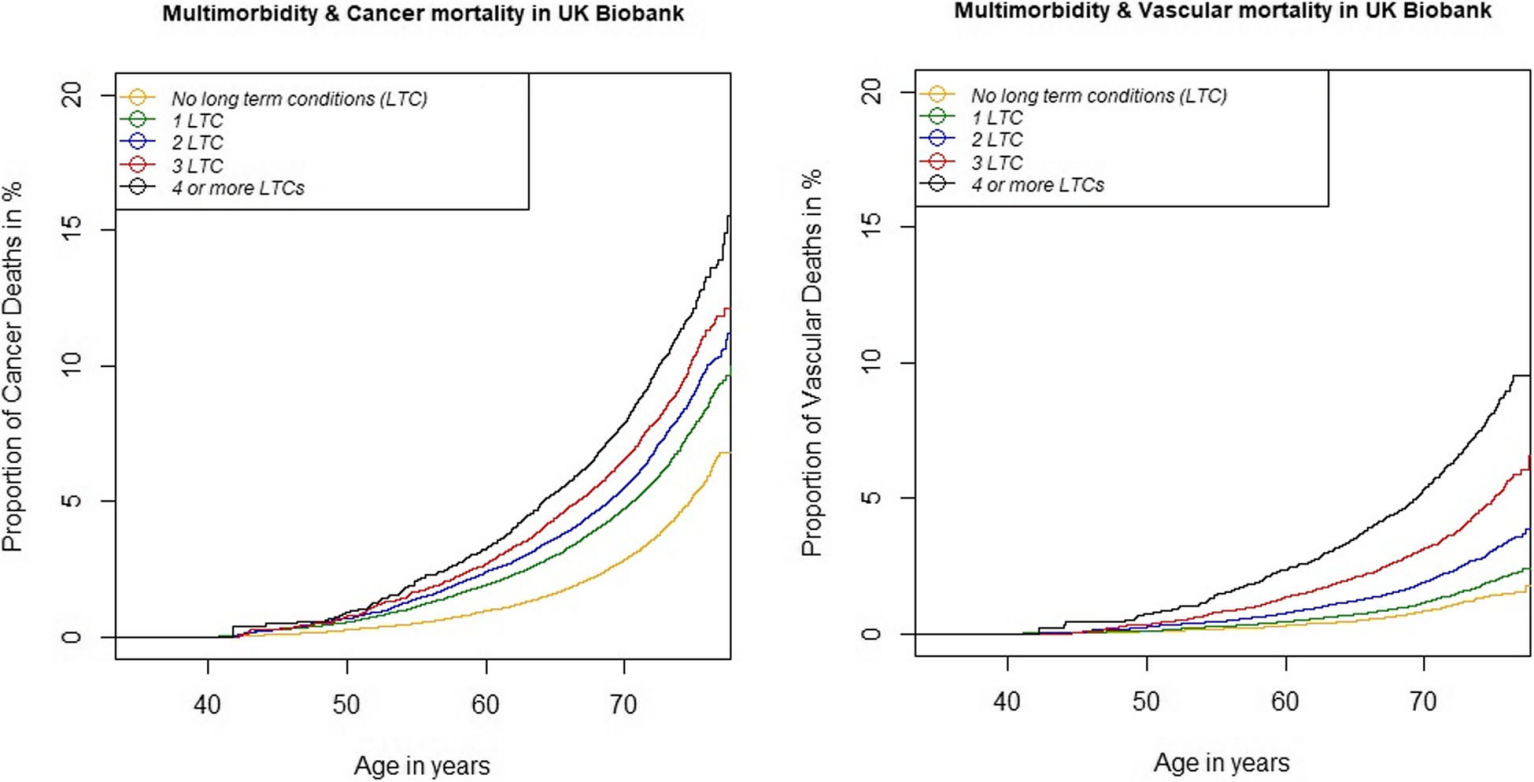
Cumulative survival plot showing the probability of cancer and vascular mortality among the UK Biobank participants with different levels of multimorbidity. *N* = 500,769 UK Biobank participants; *LTCs* long-term conditions

The majority of deaths (*N* = 8350, 58.2% of total deaths) were attributed to cancer-related causes, while vascular deaths (*N* = 2985, 20.8% of all deaths) were the second most common cause. The mean age for those who died due to cancer-related causes was 61.4 years (62.1 years for males and 60.6 years for females); the mean age for those who died due to vascular causes was 61.8 years (61.7 years for males, 61.8 years for females). Deaths due to cancer- and vascular-related causes were more frequent among participants with LTCs at baseline throughout the follow-up period, and particularly among participants with ≥ 4 LTCs (see Fig. 2). In the fully adjusted models, participants with 1 LTC were significantly more likely to die due to both cancer (HR 1.50; 95% CI 1.41-1.60) and vascular causes (HR 1.31; 95% CI 1.15-1.48), compared to participants with no LTCs (see Table 3). A dose-response relationship was observed in participants with a higher number of LTCs, who showed a higher risk of cancer and vascular mortality; with larger effect sizes observed for vascular mortality risk (Table 3). Participants with ≥ 4 LTCs were more than twice as likely to die due to cancer-related causes (HR 2.01, 95%CI 1.84-2.20) and nearly four times more likely to die due to vascular causes (HR 3.71, 95%CI 3.23-4.27), compared to participants with no LTCs. The full results for the regression models in Table 3 for cancer and vascular mortality are presented in Additional file 1: Table S3 and Table S4, respectively.

**Table 3:** Multimorbidity, cancer and vascular mortality over 7-year median follow-up: Cox’s regression analysis

Cancer mortality			
*N* = 500,769	7-year cumulative cancer mortality	Unadjusted number of events = 8350	Adjusted* (missing values *n* = 12,045; 2.4%); number of events = 8037
		Hazard ratios (95% confidence intervals)	
No LTC *N* = 172,593	1524 (0.88%)	1	1
1 LTC *N* = 163,705	2723 (1.66%)	1.56 (1.46–1.66)	1.50 (1.41–1.60)
2 LTC *N* = 95,226	2086 (2.19%)	1.80 (1.68–1.92)	1.66 (1.55–1.78)
3 LTC *N* = 43,120	1155 (2.67%)	2.04 (1.89–2.21)	1.79 (1.65–1.94)
≥ 4 LTC *N* = 26,125	862 (3.29%)	2.44 (2.25–2.66)	2.01 (1.84–2.20)
Vascular mortality			
	7-year cumulative vascular mortality	Unadjusted number of events = 2985	Adjusted* (missing values *n* = 12,045; 2.4%); number of events = 2799
		Hazard ratios (95% confidence intervals) for vascular mortality	
No LTC *N* = 172,593	429 (0.24%)	1	1
1 LTC *N* = 163,705	691 (0.42%)	1.41 (1.25–1.59)	1.31 (1.15–1.48)
2 LTC *N* = 95,226	729 (0.76%)	2.25 (1.99–2.54)	1.89 (1.67–2.14)
3 LTC *N* = 43,120	565 (1.3%)	3.58 (3.15–4.07)	2.74 (2.39–3.13)
≥ 4 LTC *N* = 26,125	571 (2.18%)	5.80 (5.11–6.59)	3.71 (3.23–4.27)

Age as the time scale for both analyses. *LTCs* long-term conditions. *Adjusted for sex, socioeconomic status based on Townsend score, smoking status, alcohol status, body mass index and physical activity levels reported at baseline

### Type of long-term conditions and clinical outcomes

When cardiometabolic multimorbidity, non-cardiometabolic multimorbidity and cancer were included as separate predictors within the same model, each had an independent and statistically significant association with all-cause mortality (see Table 4). Cardiometabolic multimorbidity also had a statistically significant association with risk of cancer and vascular mortality. The presence of multiple non-cardiometabolic LTCs (but excluding cancer) had a statistically significant association with vascular mortality, but not with cancer mortality. Previous history of cancer was associated with a statistically significant higher risk of cancer mortality but not vascular mortality.

**Table 4:** Type of long-term conditions and all-cause, cancer and vascular mortality over 7-year median follow-up: Cox’s regression analysis

Type of LTCs	*N* = 500,769. Adjusted analyses* (missing values *n* = 12,045; 2.4%)		
	Hazard ratios (95% confidence intervals) for All-cause mortality	Hazard ratios (95% confidence intervals) for cancer mortality	Hazard ratios (95% confidence intervals) for vascular mortality
No cardiometabolic conditions	1	1	1
1 LTC-cardiometabolic	1.19 (1.15–1.24)	1.04 (0.99–1.10)	1.79 (1.63–1.96)
2 LTC-cardiometabolic	1.67 (1.58–1.77)	1.15 (1.06–1.24)	3.42 (3.06–3.82)
3 LTC-cardiometabolic	2.52 (2.31–2.76)	1.23 (1.05–1.44)	7.31 (6.32–8.46)
≥ 4 LTC-cardiometabolic	3.20 (2.56–4.00)	1.67 (1.12–2.51)	8.20 (5.81–11.58)
No previous cancer	1	1	1
Presence history of Cancer	2.83 (2.71–2.95)	4.26 (4.06–4.47)	0.99 (0.87–1.13)
No non-cardiometabolic condition	1	1	1
1 LTC (excluding cancer and cardiometabolic)	1.08 (1.04–1.12)	0.99 (0.95–1.04)	1.02 (0.94–1.11)
2 LTCs (excluding cancer and cardiometabolic)	1.16 (1.10–1.22)	0.98 (0.92–1.05)	1.15 (1.03–1.29)
3 LTCs (excluding cancer and cardiometabolic)	1.25 (1.16–1.35)	0.96 (0.86–1.07)	1.29 (1.10–1.52)
4 LTCs (excluding cancer and cardiometabolic)	1.50 (1.36–1.67)	0.89 (0.75–1.05)	1.62 (1.31–1.99)

Age as the time scale. *LTC* long-term conditions, *cardiometabolic conditions* hypertension, coronary heart disease, peripheral vascular disease, atrial fibrillation, diabetes, heart failure, stroke or transient ischaemic attack. *Adjusted for sex, socioeconomic status based on Townsend score, smoking status, alcohol status, body mass index and physical activity levels reported at baseline

We considered 24 individual LTCs with the greatest individual statistically significant association with higher all-cause mortality in the whole cohort for possible combinations. These 24 LTCs were dementia (HR 5.84, 95% CI 4.11–8.31), psychoactive substance addiction (HR 4.32, 95% CI 2.50–7.44), chronic kidney disease (HR 3.61, 95% CI 3.10–4.22), alcohol addiction (HR 3.32, 95% CI 2.75–4.00), Parkinson’s disease (HR 3.10, 95% CI 2.57–3.74), heart failure (HR 2.98, 95% CI 2.44–3.64), chronic liver disease (HR 2.99, 95% CI 2.44–3.65), previous history of cancer (HR 2.83, 95% CI 2.72–2.95), chronic obstructive pulmonary disease (COPD) (HR 2.07, 95% CI 1.93–2.23), peripheral vascular disease (HR 1.95, 95% CI 1.61–2.37), schizophrenia/bipolar disorder (HR 1.72, 95% CI1.42–2.08), pernicious anaemia (HR 1.66, 95% CI 1.34–2.07), stroke/transient ischaemic attack (TIA) (HR 1.67, 95% CI 1.54–1.80), epilepsy (HR 1.65, 95% CI 1.43–1.90), diabetes (HR 1.61, 95% CI 1.53–1.70), coronary heart disease (CHD) (HR 1.60, 95% CI 1.52–1.69), bronchiectasis (HR 1.59, 95% CI 1.23–2.04), atrial fibrillation (AF) (HR 1.43, 95% CI 1.25–1.62), connective tissue disorders (HR 1.37, 95% CI 1.25–1.50), inflammatory bowel disease (HR 1.39, 95% CI 1. 20–1.62), viral hepatitis (HR 1.38, 95% CI 1.06–1.79), osteoporosis (HR 1.30, 95% CI 1.16–1.45), depression (HR 1.25, 95% CI 1.17–1.34) and hypertension (HR 1.20, 95% CI 1.16–1.25).

In the LTC = 2 category, 75 different combinations of the 24 LTCs described above were assessed for their effect size on all-cause mortality. The rest of the combinations were excluded as the number of observations was found to be less than 20. The top 10 most impactful combinations of 2 LTCs are presented in Table 5. Similarly, 43 and 25 different combinations of the aforementioned 24 LTCs were analysed for all-cause mortality risk in the LTC = 3 and LTC = ≥ 4 categories, respectively, as they met the criteria described above. The rest of the combinations were excluded as the number of observations was found to be less than 20. The top 10 most impactful combinations in these categories are shown in Table 5. For those with ≥ 4 LTCs, morbidity combinations that included hypertension, coronary heart disease, chronic kidney disease, stroke/TIA, diabetes, cancer, epilepsy, chronic obstructive pulmonary disease, depression, osteoporosis and connective tissue disorders had the greatest impact on mortality.

**Table 5:** The most impactful LTC combinations in stratified Cox’ regression analysis for mortality, for three different multimorbidity categories (based on LTC count)

Top 10 most impactful LTC combinations in each category of MM count					
Adjusted hazard ratios for all-cause mortality, when compared to reference group—no LTCs; number of deaths					
Category LTC count = 2; *N* = 95,226; total number of deaths *N* = 3555		Category 3: LTC count = 3, *N* = 43,120; total number of deaths *N* = 2213		Category 4: LTC count = ≥ 4, *N* = 26,125; total number of deaths *N* = 2025	
Cancer + bronchiectasis	9.50 (3.56–25.36); *N* = 4 deaths	Cancer + HTN + CKD	12.27 (5.50–27.39); *N* = 6 deaths	Cancer + HTN + CHD + epilepsy	7.75 (2.48–24.21); *N* = 3 deaths
Cancer + epilepsy	9.06 (5.54–14.84); *N* = 16 deaths	HTN + CKD + diabetes	11.46 (6.29–20.89); *N* = 13 deaths	HTN + CKD + CHD + diabetes	7.16 (4.20–12.22); *N* = 17 deaths
Alcohol problem + HTN	7.49 (4.23–13.28); *N* = 12 deaths	Cancer + stroke/TIA + CHD	8.16 (3.05–21.84); *N* = 4 deaths	Cancer + HTN + CHD + connective tissue disorders	6.84 (3.40–13.76); *N* = 8 deaths
Alcohol problem + depression	5.71 (2.13–15.28); *N* = 4 deaths	HTN + CKD + CHD	8.13 (3.37–19.61); *N* = 5 deaths	Cancer + HTN + depression + COPD	6.60 (3.70–11.78); *N* = 12 deaths
Epilepsy + diabetes	5.60 (1.80–17.40); *N* = 3 deaths	Cancer + connective tissue disorders + osteoporosis	8.07 (3.34–19.47); *N* = 5 deaths	Cancer + HTN + diabetes + COPD	6.38 (3.81–10.70); *N* = 16 deaths
Cancer + COPD	5.54 (3.59–8.54); *N* = 22 deaths	Cancer + diabetes + CHD	8.01 (3.78–16.93); *N* = 7 deaths	Cancer + HTN + depression + osteoporosis	5.87 (2.19–15.69); *N* = 4 deaths
Cancer + peripheral vascular disease	5.14 (1.28–20.57); *N* = 2 deaths	CHD + COPD + diabetes	7.84 (3.23–19.01); *N* = 5 deaths	Cancer + HTN + stroke/TIA + COPD	5.38 (2.68–10.83); *N* = 8 deaths
Cancer + IBD	4.98 (2.58–9.59); *N* = 9 deaths	Cancer + HTN + IBD	5.59 (1.80–17.35); *N* = 3 deaths	Cancer + HTN + diabetes + connective tissue disorders	5.03 (1.61–15.68); *N* = 16 deaths
IBD + osteoporosis	4.88 (2.02–11.75); *N* = 5 deaths	Cancer + HTN + COPD	5.55 (3.06–10.06); *N* = 11 deaths	HTN + diabetes + depression + stroke/TIA	4.87 (2.90–8.18); *N* = 19 deaths
Osteoporosis + epilepsy	4.77 (1.53–14.80); *N* = 3 deaths	Cancer + HTN + stroke/TIA	5.43 (3.62–8.14); *N* = 24 deaths	HTN + CHD + diabetes + stroke/TIA	4.64 (3.50–6.14); *N* = 63 deaths

All predictors entered individually in separate models using zero LTC group as the reference category and age as time scale; adjusted for sex, socioeconomic status based on Townsend score, smoking, alcohol consumption, body mass index and physical activity levels at baseline

*LTC* long-term condition, *HTN* hypertension, *COPD* chronic obstructive pulmonary disease, TIA transient ischaemic attack, CHD coronary heart disease, CKD chronic kidney disease, *IBD* inflammatory bowel disease. *Connective tissue disorders* myositis/myopathy, systemic lupus erythematosus, Sjogren’s syndrome/sicca syndrome, dermatopolymyositis, scleroderma/systemic sclerosis, rheumatoid arthritis, psoriatic arthropathy, dermatomyositis, polymyositis, polymyalgia rheumatica, coeliac disease

### Multimorbidity, demographics and mortality risk

**Fig. 3:**
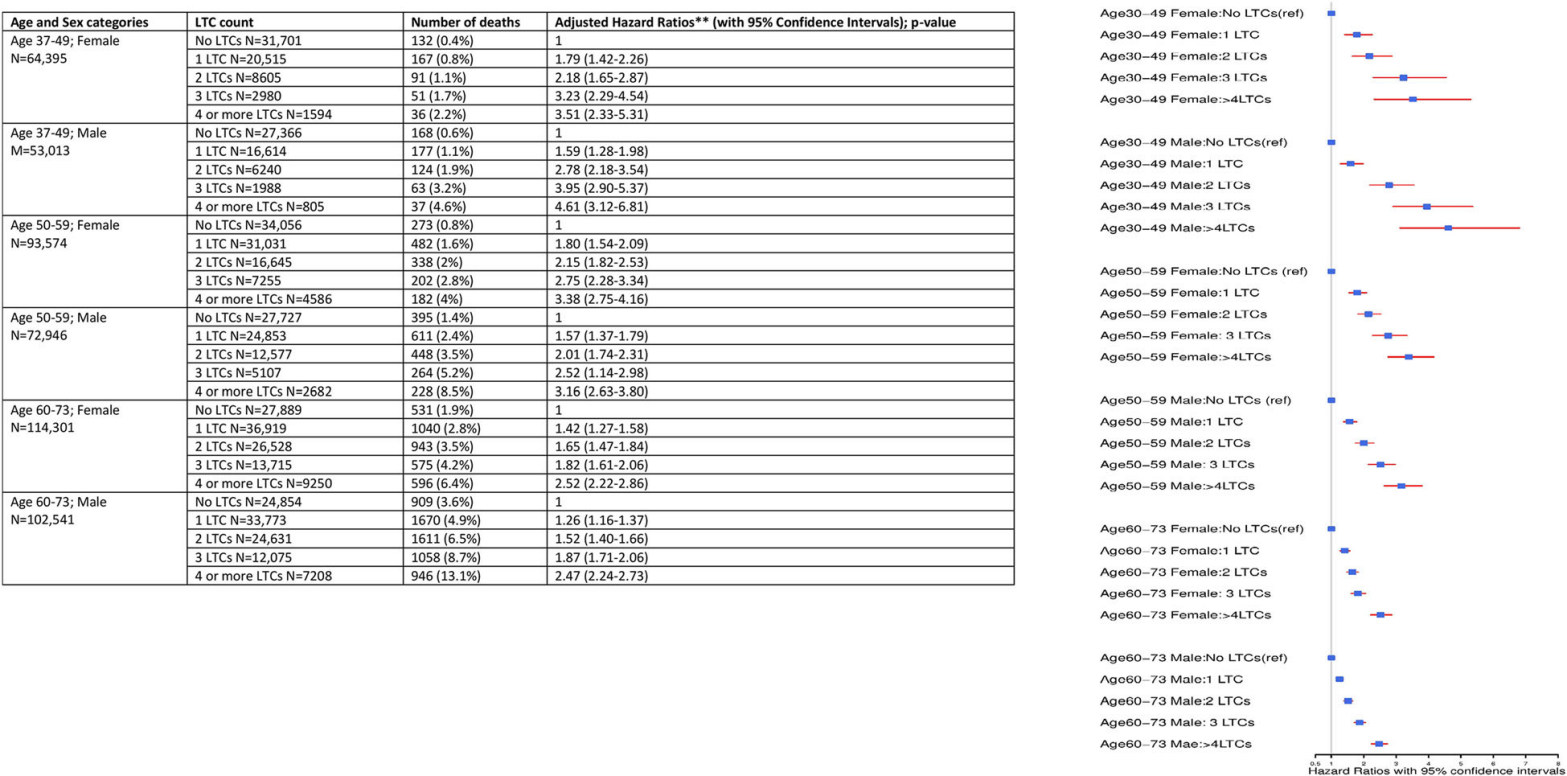
The relationship between age, sex and multimorbidity in predicting all-cause mortality. *N* = 500,769. *LTCs* long-term conditions. Two asterisks indicate the results adjusted for socioeconomic status (Townsend score), smoking status, alcohol consumption, BMI, and physical activity levels at baseline

A statistically significant interaction was observed between LTC categories and both age (*p* value < 0.0001) and sex (*p* value = 0.0019) in risk prediction of all-cause mortality. In regression models stratified by age and sex, the absolute event rate for all-cause mortality was higher for the older age group but the relative effect sizes for mortality risk with increasing number of LTCs were higher for the younger age group. Absolute mortality was highest in the older age group 60–73 years with ≥ 4 LTCs (13.1% for males and 6.4% for females) (see Fig. 3). However, the relative effect size (fully adjusted models) on all-cause mortality was lowest for adults with ≥ 4 LTCs in the older age group (HR 2.47, 95% CI 2.24-2.73 for males and HR 2.52, CI 95% 2.22-2.86 for females), compared to participants in the older age group with no LTCs. Participants in the younger age group 37–49 years with ≥ 4 LTCs had the highest relative risk of all-cause mortality (HR 4.61, 95% CI 3.12-6.81 for males and HR 3.51, 95% CI 2.33-5.31 for females), compared to participants with no LTCs in the same age group. Of note, in the younger age group (37–49 years), men were observed to have greater effect sizes of an increasing number of LTCs on all-cause mortality risk. However, in the other two age groups, the observed effect sizes were similar for both men and women.

**Fig. 4:**
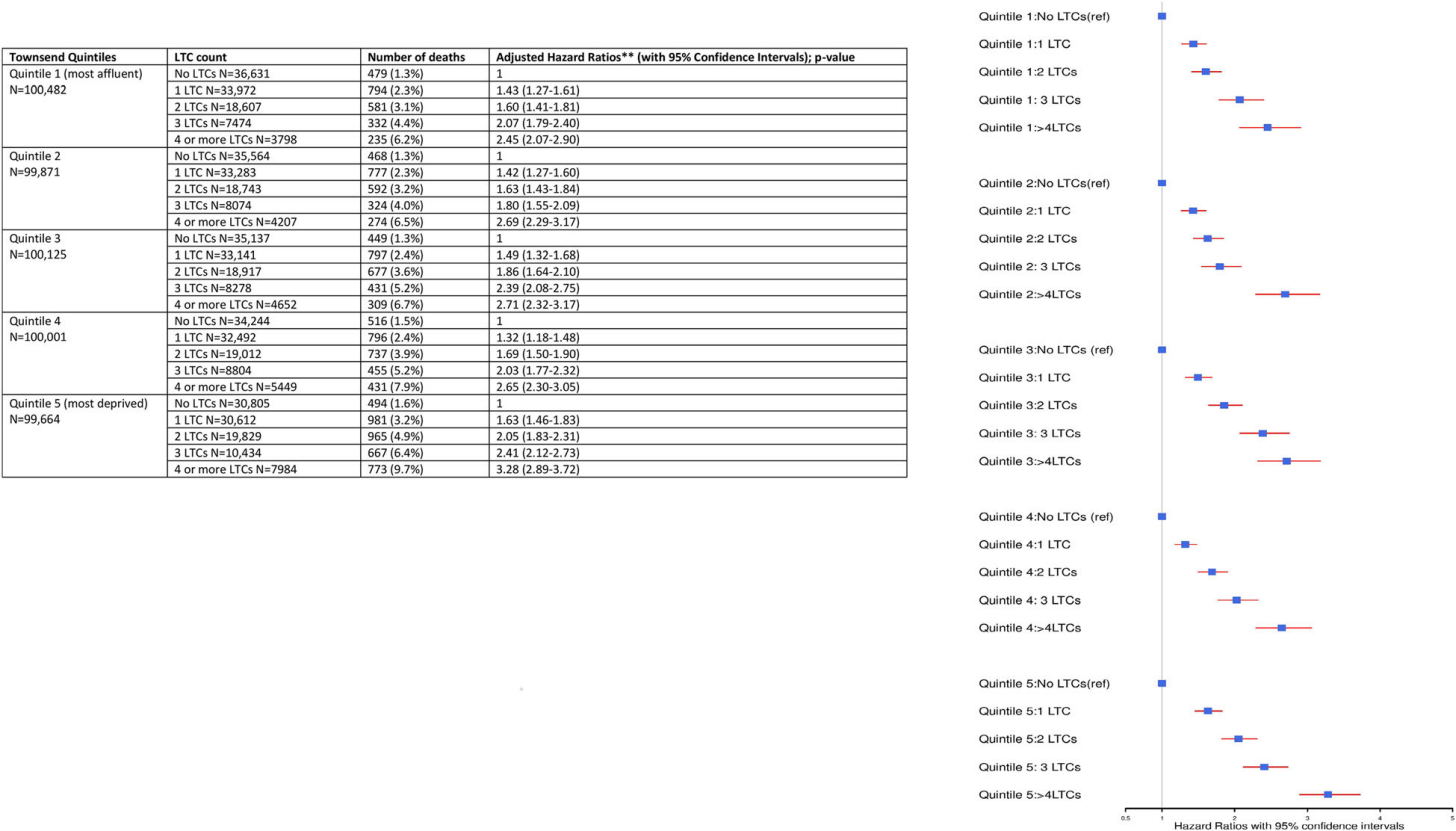
The relationship between socioeconomic status and multimorbidity in predicting all-cause mortality. *N* = 500,143. *LTCs* long-term conditions. Socioeconomic status classified based on Townsend score quintiles. Two asterisks indicate the results adjusted for age, sex, smoking status, alcohol consumption, BMI and physical activity levels at baseline

The interaction between LTC categories and socioeconomic status (based on Townsend quintiles) in risk prediction of all-cause mortality was not statistically significant (*p* value = 0.156). In stratified regression models based on socioeconomic status, a statistically significant association was observed between LTC categories and risk of all-cause mortality for all five categories of Townsend score quintiles (Fig. 4). The absolute event number of events (all-cause mortality) was noted to be highest for the most deprived participants with ≥ 4 LTCs at 9.7%. In the fully adjusted models (see Fig. 4), the relative effect size on the risk of all-cause mortality with a higher number of LTCs was consistently similar across all five socioeconomic status categories.

### Sensitivity analysis

A greater dose-response relationship was observed between a number of LTCs and risk of all-cause mortality over 7 years when LTC count was defined using previous hospitalisation records (please see Additional file 1: Table S5), instead of self-reported conditions.

## Discussion

### Summary of findings

This large prospective community cohort study involving nearly half a million middle to older aged participants demonstrates that multimorbidity has a strong dose-response relationship with all-cause mortality. In comparison to participants with no LTCs, participants with 1 LTC were nearly one and a half times more likely to die, participants with 2 LTCs were more than one and a half times more likely to die, participants with 3 LTCs were more than twice as likely to die, and participants with ≥ 4 LTCs were nearly three times more likely to die, over the median 7 years of follow-up. These results were adjusted for the effects of a broad range of potential demographic and lifestyle-related confounding factors. Importantly, a similar statistically significant relationship was observed between the number of conditions reported at baseline and the risk of cancer and vascular deaths, with greater effect sizes observed for vascular death risk. Cardiometabolic multimorbidity had a significant relationship with all-cause, cancer and vascular mortality, while non-cardiometabolic multimorbidity (excluding cancer) had a significant relationship with only all-cause and vascular mortality, but with a moderate effect size. Cancer and cardiometabolic LTCs accounted for the majority of the most impactful individual combinations of LTCs based on all-cause mortality risk. For those with ≥ 4 LTCs, morbidity combinations that included hypertension, coronary heart disease, chronic kidney disease, stroke/TIA, diabetes, cancer, epilepsy, chronic obstructive pulmonary disease and connective tissue disease had the greatest impact on mortality. In age- and sex-stratified analysis, the absolute mortality rate was higher among the older age group with a higher number of LTCs; however, the observed relative effect size between a higher number of LTCs and higher mortality risk was larger in the younger age group (37–49 years), especially among male participants. While socioeconomic status remained an independent and significant predictor of all-cause mortality, the relationship between multimorbidity and mortality risk was consistent across all categories of socioeconomic status in the stratified analysis.

### Strengths and limitations

The UK Biobank is recognised to be a high-quality resource; however, the recruited population is mostly white British and less socioeconomically deprived than the UK general population (although all strata of socioeconomic spectrum are represented). It is therefore likely that adverse lifestyle risk factors are less common than the UK average [28]. This suggests that the effect sizes for multimorbidity and mortality presented here, and the moderating effects of socioeconomic status, are likely to provide more conservative estimates than in the wider UK population. The use of self-reported health data is a potential limitation, and it was not possible to validate the presence of these self-reported long-term conditions. However, participants reported their health conditions with the support of a nurse and importantly our sensitivity analyses (repeating the analyses using health conditions based on hospitalisation records) showed the trends in results were the same with larger effect sizes, although it should be noted that the sensitivity analyses using hospitalisation records are likely to miss LTCs that are not commonly associated with hospitalisation, for example, a skin-related LTC like eczema. The large sample size is a strength of this study, as is the ability to adjust for a wide range of sociodemographic and lifestyle factors. The examination of patient characteristics in relation to the number of LTCs was cross-sectional, and therefore, temporal relationships could not be determined. There is no consensus in the literature on the best way to measure multimorbidity [29]. A simple count was deemed suitable due to the availability of data on a wide range of morbidities and the lack of evidence that alternative approaches are preferable [5]; however, this count was unweighted and we have no information on the severity of the conditions reported here. Residual confounding is likely to be a major limitation of any observational study of this kind. We have tried to minimise the effects of residual confounding by adjusting for major risk factors associated with mortality globally [30, 31] and by considering different types of LTCs and difference causes of mortality in our analysis.

### Comparison with other literature

We found a dose-response relationship between the number of LTCs and risk of all-cause mortality, as well as cancer and vascular mortality in a general population sample. A meta-analysis of 26 studies by Nunes et al. found similar effect sizes while studying the relationship between multimorbidity and all-cause mortality (HR 1.73 for ≥ 2 LTCs, HR 2.72 for ≥ 3 LTCs) in participants > 65 years of age [6]. However, while there is data to show multimorbidity is associated with lung and ovarian cancer mortality, this is the first study we know of to examine the relationship of multimorbidity with all-cause and cancer mortality [32, 33]. In our study of a general population, we found that cancer mortality risk was significantly higher with higher cardiometabolic multimorbidity (HR 1.15 for 2 LTCs, HR 1.23 for 3 LTCs, 1.67 for ≥ 4 LTCs) but unchanged with non-cardiometabolic multimorbidity that excluded a pre-existing cancer diagnosis. Previous studies conducted in populations with cancer have found an association between multimorbidity and all-cause mortality (HRs ranging from 1.1 to 5.8) [34, 35], but we could find no other studies that have examined multimorbidity and cancer mortality in a general population. We found a dose-response relationship between the presence of cardiometabolic multimorbidity (based on *N* = 7 LTCs) and all-cause (HR 1.67 for 2 LTCs, HR 2.52 for 3 LTCs, HR 3.20 for ≥ 4 LTCs) and vascular mortality (HR 3.42 for 2 LTCs, HR 7.31 for 3 LTCs, HR 8.20 for ≥ 4 LTCs). The Emerging Risk Factor Collaboration studied the effects of cardiometabolic multimorbidity (based on 3 LTCs—diabetes, stroke and myocardial infarction) in two large cohorts (including the UK Biobank) and found a similar dose-response effect on all-cause mortality risk, with slightly larger effect sizes (HRs ranging from 3.1 to 3.9 for 2 LTCs, HRs ranging from 4.9 to 6.0 for 3 LTCs) [36]. Whilst the effect of multimorbidity on vascular mortality has been studied elsewhere, it has only been studied in populations using a selective sample of pre-existing cardiometabolic conditions [37, 38]. We identified clusters of long-term conditions with the strongest association with all-cause mortality across different levels of multimorbidity. This is a key research gap highlighted in the recent Academy of Medical Sciences Report [1]. Our findings highlight the most impactful combinations of LTCs and highlight the need for further research to better understand the relationships between these conditions and how they might interact.

In the stratified regression models, we found a significant statistical interaction between both age and sex with a number of LTCs on the risk of all-cause mortality, showing an increasing number of LTCs had a greater effect size on mortality risk among younger age groups, especially men. This is the first study we know of to compare this relationship across different age groups. While socioeconomic status was a significant predictor of all-cause mortality risk, the effect of the number of LTCs on mortality risk remained similar across different socioeconomic strata, and we did not observe a significant statistical interaction between socioeconomic groups (classified on the basis of Townsend score) and number of LTCs on all-cause mortality risk. Previous research on population samples in Canada [16] and Norway [17] has found that effects of multimorbidity on mortality remained constant across different socioeconomic groups, defined on the basis of median neighbourhood income and individual educational qualifications. In a meta-analysis of 1.7 million participants from seven different high-income countries, lower socioeconomic status was found to be a risk factor of mortality, independent of other determinants of mortality such as smoking, alcohol consumption, lack of physical activity, obesity, diabetes and hypertension; however, the interaction of socioeconomic status with the presence of LTCs was not explored in this study [30].

One of the key research recommendations from the recently published NICE guidelines on multimorbidity is to develop algorithms and prediction tools for patients to predict reduced life expectancy based on multimorbidity [39]. This may inform decision making for treatment options in older people, while young people at risk can be targeted for preventative interventions. We have identified clusters of LTCs associated with the highest risk of mortality; clinicians can use this information while risk stratifying patients with multimorbidity in routine practice. Our findings suggest that the impact of multimorbidity on survival may vary significantly across different age groups with relative mortality risk higher among younger male adults with multimorbidity, hence future life expectancy algorithms using multimorbidity need to take this into account. Secondly, the majority of interventions for the management of multimorbidity to date have been targeted towards relatively older adults [40, 41], and these findings suggest there is a need for future research to develop interventions for managing multimorbidity in middle-aged populations and to explore whether multimorbidity should be noted as a risk factor within cancer referral pathways.

## Conclusion

In our study of middle to older aged participants recruited from the general population, multimorbidity was consistently associated with higher all-cause, cancer and vascular mortality, even after adjusting for the effects of lifestyle and demographic factors. Cardiometabolic multimorbidity was noted to have a consistent association with all three clinical outcomes considered, while cancer and cardiometabolic conditions were featured in almost all the most impactful combinations of LTCs for mortality risk. Type of LTCs, as opposed to a number, may have an important role in understanding the relationship between multimorbidity and mortality. Younger participants, especially men, were observed to have a relatively higher risk of mortality with increasing number of LTCs. Further research is needed to study the impact and management of multimorbidity in middle-aged adults, as they may be at higher risk of early death.

## Additional file

Additional file 1: The file contains additional information and analysis, such as a list of LTCs, full results of regression models, sensitivity analysis with a list of LTCs captured by Hospital Episode Statistics. **Table S1.** List of self-reported long-term conditions considered for multimorbidity count. **Table S2.** Multimorbidity and all-cause mortality: Cox’s regression analysis. *N* = 500,771. **Table S3.** Multimorbidity and cancer mortality: Cox’s regression analysis. *N* = 500,771. **Table S4.** Multimorbidity and vascular mortality: Cox’s regression analysis. *N* = 500,771. **Table S5.** Comparison of LTCs (self-report vs. HES) in the prediction of all-cause mortality over 7-year median follow-up: Cox’s regression analysis. (DOCX 29 kb)

## Abbreviations

AF: Atrial fibrillation
Cardiometabolic conditions: Hypertension, coronary heart disease, peripheral vascular disease, atrial fibrillation, diabetes, heart failure, stroke or transient ischaemic attack
CHD: Coronary heart disease
CKD: Chronic kidney disease
Connective tissue disorders: Myositis/myopathy, systemic lupus erythematosus, Sjogren’s syndrome/sicca syndrome, dermatopolymyositis, scleroderma/systemic sclerosis, rheumatoid arthritis, psoriatic arthropathy, dermatomyositis, polymyositis, polymyalgia rheumatica, coeliac disease
COPD: Chronic obstructive pulmonary disease
HR: Hazard ratio
HTN: Hypertension
IBD: Inflammatory bowel disease
LTCs: Long-term conditions
SD: Standard deviation
TIA: Transient ischaemic attack
