# Supplementary material for: Relationship between multimorbidity, demographic factors and mortality: findings from the UK Biobank cohort
Source: BMC Med. 2019 Apr 10;17:74. doi: 10.1186/s12916-019-1305-x (PMC6456941; doi:10.1186/s12916-019-1305-x)
Supplement: Supplementary file 1 — The file contains additional information and analysis, such as a list of LTCs, full results of regression models, sensitivity analysis with a list of LTCs captured by Hospital Episode Statistics. Table S1. List of self-reported long-term conditions considered for multimorbidity count. Table S2. Multimorbidity and all-cause mortality: Cox’s regression analysis. N = 500,771. Table S3. Multimorbidity and cancer mortality: Cox’s regression analysis. N = 500,771. Table S4. Multimorbidity and vascular mortality: Cox’s regression analysis. N = 500,771. Table S5. Comparison of LTCs (self-report vs. HES) in the prediction of all-cause mortality over 7-year median follow-up: Cox’s regression analysis. (DOCX 29 kb) [file 12916_2019_1305_MOESM1_ESM.docx]

**Title: Relationship of Multimorbidity, Demographic Factors and Mortality: Findings from UK Biobank Cohort**

**Additional file 1**

**Table S1 List of self-reported long-term conditions considered for multimorbidity count**

| **Long term condition grouping** | **Conditions included as reported by participants** |
| --- | --- |
| 1. Painful conditions | Back pain  Joint pain  Back pain  Joint pain  Headaches (not migraine)  Sciatica  Plantar fasciitis  Carpal tunnel syndrome  Fibromyalgia  Arthritis  Shingles  Disc problem  Prolapsed disc/slipped disc  Spine arthritis/spondylitis  Ankylosing spondylitis  Back problem  Osteoarthritis  Gout  Cervical spondylosis  Trigeminal neuralgia  Disc degeneration  Trapped nerve/compressed nerve |
| 1. Hypertension | Hypertension  Essential Hypertension |
| 1. Depression | Depression  Postnatal Depression |
| 1. Asthma | Asthma |
| 1. Atrial Fibrillation | Atrial Fibrillation |
| 1. Coronary Heart Disease | Heart attack/Myocardial Infarction  Angina |
| 1. Dyspepsia | Gastro-oesophageal reflux (GORD)/gastric reflux  Oesophagitis /Barrett's oesophagus  Gastric stomach ulcers  Gastric erosions/gastritis  Duodenal ulcer  Dyspepsia/indigestion  Hiatus hernia  Helicobacter pylori |
| 1. Diabetes | Diabetic nephropathy  Diabetic neuropathy/ulcers  Diabetes  Type 1 diabetes  Type 2 diabetes  Diabetic eye disease |
| \| 1. Thyroid disorders \| \| --- \| | Thyroid problem (not cancer)  Hyperthyroidism/thyrotoxicosis  Hypothyroidism/myxoedema  Grave’s disease  Thyroid goitre  Thyroiditis |
| 1. Connective tissue disorders | Myositis/myopathy  Systemic Lupus Erythematosus  Connective tissue disorder  Sjogrens syndrome/sicca syndrome  Dermatopolymyositis  Scleroderma/systemic sclerosis  Rheumatoid arthritis  Psoriatic arthropathy  Dermatomyositis  Polymyositis  Polymyalgia Rheumatica  Malabsorption/coeliac disease |
| 1. Chronic Obstructive Pulmonary Disease (COPD) | COPD/chronic obstructive airways disease  Emphysema/chronic bronchitis  Emphysema |
| 1. Anxiety | Anxiety/panic attacks  Nervous breakdown  Post-traumatic stress disorder  Obsessive compulsive disorder  Stress  Insomnia  Psychological/psychiatric problem |
| 1. Irritable bowel syndrome | Irritable bowel syndrome |
| 1. Alcohol problems | Alcohol dependency  Alcoholic liver disease/alcoholic cirrhosis |
| 1. Other psychoactive substance abuse | Opioid dependency  Other substance abuse/dependency |
| 1. Treated constipation | Constipation |
| 1. Stroke/Transient Ischaemic Attack (TIA) | Stroke  TIA  Subarachnoid haemorrhage  Brain haemorrhage  Ischaemic stroke |
| 1. Chronic kidney disease | Polycystic kidney  Diabetic nephropathy  Renal/kidney failure  Renal failure requiring dialysis  Renal failure not requiring dialysis  Kidney nephropathy  Immunoglobulin A (IgA) nephropathy |
| 1. Diverticular disease | Diverticular disease  Diverticulitis |
| 1. Peripheral vascular disease | Peripheral vascular disease  Leg claudication/intermittent claudication |
| 1. Heart failure | Cardiomyopathy  Hypertrophic cardiomyopathy  Heart failure/pulmonary oedema |
| 1. Prostate disorders | Prostate problem (not cancer)  Enlarged prostate  Benign prostatic hypertrophy |
| 1. Glaucoma | Glaucoma |
| 1. Epilepsy | Epilepsy |
| 1. Dementia | Dementia  Alzheimer’s disease  Cognitive impairment |
| 1. Schizophrenia/bipolar disorder | Schizophrenia  Mania/  Bipolar disorder  Manic depression |
| 1. Psoriasis/eczema | Eczema  Dermatitis  Psoriasis |
| 1. Inflammatory Bowel Disease | Inflammatory Bowel Disease  Crohn’s disease  Ulcerative colitis |
| 1. Migraine | Migraine |
| 1. Chronic sinusitis | Chronic sinusitis |
| 1. Anorexia or bulimia | Anorexia  Bulimia  Other eating disorders |
| 1. Bronchiectasis | Bronchiectasis |
| 1. Parkinson’s disease | Parkinson’s disease |
| 1. Multiple Sclerosis | Multiple Sclerosis |
| 1. Viral Hepatitis | Infective/viral hepatitis  Hepatitis B  Hepatitis C  Hepatitis D  Hepatitis E |
| 1. Chronic Liver disease | Oesophageal varices  Non infective hepatitis  Liver failure/cirrhosis  Primary biliary cirrhosis |
| 1. Osteoporosis | Osteoporosis |
| 1. Chronic fatigue syndrome | Chronic fatigue syndrome |
| 1. Endometriosis | Endometriosis |
| 1. Meniere’s disease | Meniere’s disease |
| 1. Pernicious Anaemia | Pernicious Anaemia |
| 1. Polycystic ovary | Polycystic ovary |
| 1. Cancer | Lifetime diagnosis |

**Table S2 Multimorbidity and all-cause mortality: Cox’s regression analysis. N=500,771**

| **Predictor variables** | **Categories for predictor variables** | **Adjusted Hazard Ratio with 95% Confidence Intervals ***  **(Missing values n=12 045, 2.4%)** | **p-value** |
| --- | --- | --- | --- |
| LTC categories | No LTCs (reference) | 1 |  |
|  | 1 LTC | 1.46 (1.38-1.54) | <0.01 |
|  | 2 LTC | 1.77 (1.68-1.87) | <0.01 |
|  | 3 LTC | 2.14 (2.01-2.28) | <0.01 |
|  | 4 or more LTC | 2.79 (2.61-2.98) | <0.01 |
| Sex | Female (reference) | 1 |  |
|  | Male | 1.86 (1.80-1.93) | <0.01 |
| Townsend score (continuous) |  | 1.04 (1.037-1.048) | <0.01 |
| Smoking | Never Smoked (reference) | 1 |  |
|  | Current or Previous Smoker | 1.54 (1.49-1.60) | <0.01 |
| Physical Activity | High (reference) | 1 |  |
|  | Medium | 1.37 (1.26-1.48) | <0.01 |
|  | Low | 1.96 (1.76-2.17) | <0.01 |
|  | None | 2.27 (2.07-2.50) | <0.01 |
| Alcohol consumption frequency | Never or special occasions only (reference) | 1 |  |
|  | 1-3 times/month | 0.81 (0.76-0.86) | <0.01 |
|  | 1-4 times/week | 0.78 (0.74-0.81) | <0.01 |
|  | Daily or almost daily | 0.82 (0.78-0.86) | <0.01 |
| Body mass index | 18.5-25 (reference) | 1 |  |
|  | <18.5 | 1.04 (1.037-1.048) | <0.01 |
|  | 25-30 | 0.83 (0.79-0.86) | <0.01 |
|  | 30-35 | 0.86 (0.82-0.91) | <0.01 |
|  | 35-40 | 0.93 (0.86-0.91) | 0.04 |
|  | >40 | 1.12 (1.01-1.24) | 0.03 |

LTC=Long-term conditions; BMI=Body Mass Index; Age as time scale *Adjusted for sex, socioeconomic status based on Townsend score, smoking status, alcohol status, BMI, and physical activity

**Table S3 Multimorbidity and cancer mortality: Cox’s regression analysis. N=500,771**

| **Predictor variables** | **Categories for predictor variables** | **Adjusted Hazard Ratio with 95% Confidence Intervals ***  **(Missing values n=12 045, 2.4%)** | **p-value** |
| --- | --- | --- | --- |
| LTC categories | No LTCs (reference) | 1 |  |
|  | 1 LTC | 1.50 (1.41-1.60) | <0.01 |
|  | 2 LTC | 1.66 (1.55-1.78) | <0.01 |
|  | 3 LTC | 1.79 (1.65-1.94) | <0.01 |
|  | 4 or more LTC | 2.01 (1.84-2.20) | <0.01 |
| Sex | Female (reference) | 1 |  |
|  | Male | 1.39 (1.33-1.45) | <0.01 |
| Townsend score (continuous) |  | 1.03 (1.02-1.03) | <0.01 |
| Smoking | Never Smoked (reference) | 1 |  |
|  | Current or Previous Smoker | 1.56 (1.49-1.63) | <0.01 |
| Physical Activity | High | 1 |  |
|  | Medium | 1.31 (1.19-1.45) | <0.01 |
|  | Low | 1.67 (1.46-1.91) | <0.01 |
|  | None | 1.81 (1.60-2.05) | <0.01 |
| Alcohol consumption frequency | Never or special occasions only (reference) | 1 |  |
|  | 1-3 times/month | 0.86 (0.79-0.94) | <0.01 |
|  | 1-4 times/week | 0.83 (0.78-0.88) | <0.01 |
|  | Daily or almost daily | 0.83 (0.78-0.89) | <0.01 |
| Body mass index | 18.5-25 (reference) | 1 |  |
|  | <18.5 | 1.52 (1.17-1.98) | <0.01 |
|  | 25-30 | 0.88 (0.83-0.93) | <0.01 |
|  | 30-35 | 0.90 (0.84-0.96) | <0.01 |
|  | 35-40 | 0.92 (0.83-1.01) | 0.09 |
|  | >40 | 0.89 (0.76-1.05) | 0.16 |

LTC=Long-term conditions; BMI=Body Mass Index; Age as time scale *Adjusted for sex, socioeconomic status based on Townsend score, smoking status, alcohol status, BMI, and physical activity

**Table S4** **Multimorbidity and vascular mortality: Cox’s regression analysis. N=500,771**

| **Predictor variables** | **Categories for predictor variables** | **Adjusted Hazard Ratio with 95% Confidence Intervals ***  **(Missing values n=12 045, 2.4%)** | **p-value** |
| --- | --- | --- | --- |
| LTC categories | No LTCs (reference) | 1 |  |
|  | 1 LTC | 1.31 (1.15-1.48) | <0.01 |
|  | 2 LTC | 1.89 (1.67-2.14) | <0.01 |
|  | 3 LTC | 2.74 (2.39-3.13) | <0.01 |
|  | 4 or more LTC | 3.71 (3.23-4.27) | <0.01 |
| Sex | Female (reference) | 1 |  |
|  | Male | 3.57 (3.26-3.89) | <0.01 |
| Townsend score (continuous) |  | 1.06 (1.05-1.07) | <0.01 |
| Smoking | Never Smoked (reference) | 1 |  |
|  | Current or Previous Smoker | 1.59 (1.46-1.72) | <0.01 |
| Physical Activity | High | 1 |  |
|  | Medium | 1.31 (1.09-1.57) | <0.01 |
|  | Low | 2.06 (1.64-2.58) | <0.01 |
|  | None | 2.44 (1.98-3.00) | <0.01 |
| Alcohol consumption frequency | Never or special occasions only (reference) | 1 |  |
|  | 1-3 times/month | 0.73 (0.63-0.85) | <0.01 |
|  | 1-4 times/week | 0.72 (0.65-0.79) | <0.01 |
|  | Daily or almost daily | 0.74 (0.66-0.83) | <0.01 |
| Body mass index | 18.5-25 (reference) | 1 |  |
|  | <18.5 | 2.33 (1.52-3.57) | <0.01 |
|  | 25-30 | 0.96 (0.86-1.06) | 0.42 |
|  | 30-35 | 1.09 (0.97-1.22) | 0.14 |
|  | 35-40 | 1.34 (1.15-1.56) | <0.01 |
|  | >40 | 1.92 (1.58-2.34) | <0.01 |

LTC=Long-term conditions; BMI=Body Mass Index; Age as time scale *Adjusted for sex, socioeconomic status based on Townsend score, smoking status, alcohol status, BMI, and physical activity

**Table S5 Comparison of LTCs (self-report vs. HES) in prediction of all-cause mortality over 7 year median follow-up: Cox’s regression analysis.**

| N=500,769 | **Unadjusted. Number of events=14348** | **Adjusted ***  **(Missing values n=12,045, 2.4%); Number of events=13570** |
| --- | --- | --- |
| Model with self-reported LTC | | |
| Hazard Ratios (95% Confidence intervals); p-value | | |
| No LTCs  N=172,593  (34.5%) | 1 | 1 |
| **1 LTC**  **N=163,705**  **(60.3%)** | 1.83 (1.74-1.92); <0.0001 | 1.47 (1.40-1.55); <0.0001 |
| 2 LTCs  N=95,226 (19%) | 2.72 (2.58-2.87); <0.0001 | 1.81 (1.71-1.91); <0.0001 |
| **3 LTCs**  **N=43,120**  **(8.6%)** | 3.78 (3.56-4.00); <0.0001 | 2.20 (2.07-2.35); <0.0001 |
| **≥4 LTCs**  **N=26,125**  **(5.2%)** | 5.78 (5.45-6.13); <0.0001 | 2.89 (2.70-3.08); <0.0001 |
| Model with LTC defined on the basis of previous hospitalization records only | | |
| Hazard Ratios (95% Confidence intervals); p-value | | |
| **N=500,762** | Unadjusted. Number of events=14347 | Adjusted *  (Missing values n=13,569, 2.4%); Number of events=12052 |
| **No LTCs**  **N=321,950 (65.7%)** | 1 | 1 |
| **1 LTC N=96,339 (19.2%)** | 1.69 (1.62-1.77); <0.0001 | 1.59 (1.52-1.66); <0.0001 |
| **2 LTC N=40,400 (8.1%)** | 2.24 (2.13-2.36); <0.0001 | 1.96 (1.85-2.06); <0.0001 |
| **3 LTC N=18,685 (3.7%)** | 3.03 (2.85-3.22); <0.0001 | 2.45 (2.30-2.61); <0.0001 |
| **≥4 LTCs N=16,188 (3.2%)** | 5.26 (5.00-5.54); <0.0001 | 3.70 (3.49-3.92); <0.0001 |

LTC=Long-term conditions; BMI=Body Mass Index; Age as time scale *Adjusted for sex, socioeconomic status based on Townsend score, smoking status, alcohol status, BMI, and physical activity
